# Management of chronic myelogenous leukemia with COVID-19 and hepatitis B

**DOI:** 10.3389/fonc.2023.1217023

**Published:** 2023-08-03

**Authors:** Tian Yu, Weiming Li, Tao Yu

**Affiliations:** ^1^ Department of Hematology, Affiliated Renhe Hospital of China Three Gorges University, Yichang, China; ^2^ College of Basic Medical Sciences, China Three Gorges University, Yichang, China; ^3^ Department of Hematology, Union Hospital, Tongji Medical College, Huazhong University of Science and Technology, Wuhan, China

**Keywords:** chronic myelogenous leukemia, tyrosine kinase inhibitors, coronavirus disease 2019, hepatitis B, management

## Abstract

The application of immunosuppressive agents and targeted drugs has opened a novel approach for the treatment of hematological tumors, and the application of tyrosine kinase inhibitors for the treatment of chronic myeloid leukemia is one of the landmark breakthroughs that has considerably improved the prognosis of CML patients. However, with the extensive use of TKI, the co-infection of CML patients has become increasingly apparent, especially regarding infectious diseases such as hepatitis B and COVID-19. The underlying mechanism may be related to the inhibition of the immune function by TKI. Poor management, including disease progression due to the infectious disease or TKI dose reduction or discontinuation, may lead to adverse clinical outcomes and can even be life-threatening. Therefore, this review principally provides an overview of the pathogenesis and standardized management principles of CML patients with comorbid COVID-19 or hepatitis B in order to improve clinicians’ awareness of the risks so as to more effectively diagnose and treat CML and improve the survival rate and quality of life of patients. In the past two decades, owing to the advent of imatinib, chronic myeloid leukemia (CML) has transformed into a chronic controllable disease, and even treatment-free remission can be anticipated. Earlier studies have indicated that tyrosine kinase inhibitor (TKI) exerts a peculiar inhibitory effect on the body’s immune function. Therefore, with the widespread application of TKI, more and more attention has been paid to the comorbidity of infectious diseases in CML patients, especially in patients with progressive disease or non-remission. Despite some studies revealing that the proportion and severity of SARS-CoV-2 infection in CML patients receiving TKI treatment are lower than in patients with other hematological malignancies, CML patients with stable disease are still recommended to be vaccinated against SARS-CoV-2, while TKI may or may not be discontinued. Meanwhile, the management of CML patients during the epidemic of coronavirus disease 2019 (COVID-19) still necessitates further discussion. This article also provides an overview of TKI-related hepatitis B reactivation. If not managed, patients may face adverse consequences such as hepatitis B reactivation-related hepatitis, liver failure, and progression of CML after forced withdrawal of medication. Therefore, this review aimed to comprehensively describe the management of CML patients with comorbid COVID-19, the pathogenesis of hepatitis B reactivation, the indicated population for prophylactic antiviral therapy, the time of antiviral drug discontinuation, and drug selection.

## 1 CML complicated with COVID-19

On February 11, 2020, the World Health Organization (WHO) named the disease caused by severe acute respiratory syndrome coronavirus 2 (SARS-CoV-2) infection as COVID-19. On 12 March of the same year, the outbreak was considered a global pandemic. Given that the majority of patients with hematological malignancies are immunocompromised, the infection rate and mortality rate of SARS-CoV-2 are high in this population. However, studies have signaled that TKIs have antiviral and immune-regulating abilities, which may reduce the incidence of COVID-19 infection (1–3). Even so, there is an urgent need to develop an appropriate risk management strategy for CML patients to manage the SARS-CoV-2 pandemic.

### 1.1 Morbidity and mortality of CML with COVID-19

CML accounts for 15% of adult leukemia, with a global incidence of 1.6/100,000 to 2/100,000, and a incidence of 0.36/100,000 to 0.55/100,000 in China (4, 5). Li et al. (6) collected data from 530 CML patients with COVID-19 in Hubei Province at the beginning of the epidemic in 2020, and the prevalence of COVID-19 was estimated to be 0.9%, which was significantly higher than that of the overall population during the same period (0.9% vs. 0.1%), yet significantly lower than that of hematology and oncology patients (0.9% vs. 10%). Another Dutch study also observed that the ratio of SARS-CoV-2 infection in CML patients was marginally higher than that in the general population (0.7% vs 0.3%) (7). At the same time, Breccia et al. (8)collected clinical information on 8665 CML patients, of which 217 were SARS-CoV-2-positive (2.5%), which was comparable to the infection rate of the general Italian population during the same period (2%-3%), and the mortality rate of CML patients was 0.13%. Compared with other malignant hematological diseases (2.04%), the mortality rate of CML seems to be lower.

The outbreak of SARS-CoV-2 Omicron (BA.5.2) began in China after December 2022 (9). A study investigated the epidemiological characteristics of this strain in CML patients by conducting a questionnaire survey for 1121 CML patients in the Hubei and Henan provinces and determined that 74% of patients developed SARS-CoV-2 infection, which was lower than the official COVID-19 infection rate of >80% in the Hubei province and 89% in the Henan province. Among these patients, 4% were asymptomatic, but only 2% (n=22) required hospitalization, with 5 mild, 14 moderate, and 3 severe cases. Moreover, five patients required respiratory assistance, three were admitted to the intensive care unit, and one (with progressive disease) died from COVID-19.The study found that age>=65 years, higher education level and receiving imatinib for CML are more susceptible to SARS-CoV-2 infection. The authors suggest that the mechanism of differences between TKIs may be related to different immune effects. Compared with imatinib, dasatinib and nilotinib may play a more significant role in resisting SARS-CoV-infection and regulating cytokine release syndrome (10).

### 1.2 Management of CML patients with COVID-19

#### 1.2.1 Can CML patients be vaccinated against COVID-19?

Experts’consensus on severe acute respiratory syndrome coronavirus-2 vaccination in adult patients with hematological diseases in China(2022) (11) recommends that those patients with CML whose condition remains stable after BCR::ABL1 TKI monotherapy maintenance therapy can be vaccinated against COVID-19. Stable disease means that CML does not progress to accelerated phase and blast crisis. Bonifacio et al. (12) conducted a cross-sectional study in 564 CML patients with COVID-19 from five hematologic centers in Italy and noted that the positive rate of lgM/lgG was similar to that of the general population (2%), regardless of the administration of TKI. Additionally, Claudiani et al. (13) validated that the vaccine response rate in CML patients was similar to that in the general population. Yang et al. (14) investigated 335 CML patients vaccinated with the SARS-CoV-2 vaccine and found that the incidence of adverse reactions was 19.1%, which was comparable to that of the healthy population in China (19.0%-48.3%). In addition, the study also exposed that the occurrence of adverse reactions was not significantly correlated with the type of TKI and patient characteristics. Therefore, SARS-CoV-2 vaccination is recommended for CML patients with stable disease.

#### 1.2.2 Which CML patients are not eligible for the COVID-19 vaccine?

Experts’consensus on severe acute respiratory syndrome coronavirus-2 vaccination in adult patients with hematological diseases in China (2022) (11) proposes that vaccination should be postponed in the following patients: 1) patients with progressive or uncontrolled diseases. 2) patients with abnormal routine blood tests, thrombocytopenia (< 50×10^9^/L), and neutropenia (< 1.0×10^9^/L). 3) Patients contraindicated for the vaccine, as described in the Guidelines of vaccination for COVID-19 vaccines in China (First edition) issued by the National Health Commission of the People’s Republic of China (15).

#### 1.2.3 Should TKIs be discontinued in CML patients with COVID-19?

The European Conference on Infections in Leukemia pointed out that non-chemotherapy targeted drugs, such as TKIs, should not be discontinued even in patients with COVID-19 (16). CANDID (Cml AND covID), initiated by the International Cml Foundation (iCMLf), enrolled 1050 CML patients infected with SARS-CoV-2, and the results showed that the severity and mortality rate was not related to the type of TKI, TKI treatment regimen, or dose. Interestingly, the proportion of TKI interruption in severe or critical patients was significantly higher (17). In addition, earlier studies have established that TKIs exert antiviral effects and alleviate inflammatory storms (1–3). Tanaka et al. (18) described that patients treated with imatinib showed selective depletion of effector T reg (eT reg) cells and a significant increase in effector/memory CD8+ T cells after achieving complete molecular remission (CMR), but not in non-CMR patients. Therefore, CML patients with COVID-19 are recommended to continue their TKI regimen.

#### 1.2.4 Does SARS-CoV-2 infection cause molecular relapse in patients with TFR?

Saußele et al. (19) collected the molecular remission status of 74 CML patients in treatment-free remission before, during, and after at least 6 months following SARS-CoV-2 infection, and the results revealed no statistically significant difference in the severity of COVID-19 symptoms between patients on TFR and TKI treatment (12.6% vs. 12%, P < 0.05). p=0.87). Besides, there was also no evidence of an increased risk of TFR loss after SARS-CoV-2 infection. Thus, SARS-CoV-2 infection may not be the cause of molecular relapse in CML patients who achieved TFR.

#### 1.2.5 The prescription principle in CML patients with COVID-19

The treatment principle of CML patients infected with COVID-19 is similar to non-covid-infected CML patients, and CML patients can be diagnosed and treated according to the treatment guidelines formulated by the National Health Commission of the People’s Republic of China. However, it is worthwhile emphasizing that overdoses of antipyretic drugs such as ibuprofen and acetaminophen and the Lianhua Qingwen capsule, as well as co-administration with Western medicine, should be avoided. *In vitro* experiments demonstrated that imatinib inhibited the O-glucuronidation of p-aminophenol. Notably, a febrile patient in a clinical trial was reportedly treated with acetaminophen and died of acute liver failure 11 days after receiving imatinib treatment. Therefore, caution is warranted in patients taking imatinib and antipyretics containing acetaminophen, ibuprofen, or diclofenac sodium suppository (20). For instance, Paxlovid contains ritonavir, a CYP3A4 enzyme inhibitor, and patients given TKI combined with anti-COVID-19 drugs should be strictly monitored for side effects. The blood drug concentration can be measured in qualified centers, and the dose of TKI should be reduced if necessary.

## 2 CML complicated with hepatitis B virus infection

In 2006, the US FDA updated the information on imatinib, dasatinib, and nilotinib to emphasize the risk of hepatitis B virus reactivation (21). Hepatitis B reactivation occurred in some patients, which subsequently fulminated into hepatitis and liver failure, resulting in an extremely poor prognosis. Therefore, it is critical to weigh the benefits of the treatment of CML patients and the risk of hepatitis B reactivation.

### 2.1 The reactivation rate of HBV in CML patients

In recent years, cases of TKI-related hepatitis B reactivation have been increasing. Prior studies conducted in Taiwan and Korea exposed that the reactivation rate of HBsAg-positive patients treated with TKIs, including imatinib, dasatinib, and nilotinib, was 26%-44.1% (See **Table 1** for details) (22–25).

**Table 1:** The effects of different TKIs on the outcomes of patients with chronic myelogenous leukemia complicated with hepatitis B.

Author	Number of cases	HBV status	Proportion of HBVr (antiviral vs. control)	Median time to HBV reactivation from CML diagnosis	Medication
Uhm (2018) (22)	N=76	HBsAg(+)	0% (0/23) vs 26% (12/46)	22.1 mo	Imatinib: 7; dasatinib: 2; nilotinib: 1; radotinib: 1; Discontinued TKI therapy: 1.
Kim (2012) (23)	N=43	HBsAg(+)	0% (0/9) vs 44.1% (15/34)	9.3 mo	Imatinib: 12; dasatinib: 2; nilotinib:1.
Wang (2019) (24)	N=19	HBsAg(+)	0% (0/6) vs 38.5% (5/13)	11 mo	
	N=123	HBsAg(-)	0%		
Sorà (2017) (25)	N=11	HBsAg(-)	0%		Imatinib: 7; nilotinib: 4.

### 2.2 The pathogenesis of HBVr in CML

On the one hand, imatinib and nilotinib have been reported to inhibit T cell-mediated immune response. On the other hand, dasatinib can restrain Src family LCK and T cell function and inhibit CD8+T cell proliferation (26, 27). Recent studies have validated that TKIs can lower the amount of immunoglobulin (28, 29). Li et al. (26) reported 2 cases of hepatitis B reactivation after achieving MMR or CCyR in patients treated with imatinib, which is in line with the findings of Ikeda et al. (30). At the same time, Mothy et al. (31) evinced that CML patients who responded to imatinib were able to recover the function of plasmacytoid dendritic cells, which may be related to immune reconstruction due to hepatitis B reactivation. Lastly, Lai et al. (26) reported a case of hepatitis B reactivation 5 months before achieving CCyR with nilotinib, which may be associated with the different immune recovery pathways of the imatinib.

### 2.3 Management of CML patients with HBVr

#### 2.3.1 Risk assessment

The American Gastroenterological Association (AGA), Asian Pacific Association for the Study of the Liver (APASL), and European Association for the Study of the Liver (EASL) recommend stratifying individuals at high risk of hepatitis B reactivation according to HBV marker status and the type of immunosuppressive agents; the patients should be classified into three risk levels according to HBV-related morbidity, namely low (<1%), moderate (1%-10%), and high (>10%). According to the WHO, the global prevalence of hepatitis B in the general population in 2019 was 3.8%. It is estimated that the prevalence rate in China is 6.1%, and there are about 86 million hepatitis B carriers (32).APASL recommends HBsAg (+) patients receiving TKI be classified as high-risk, whereas HBsAg (-) patients are classified as low-risk. Contrastingly, AGA considers these patients to be in the moderate risk group, regardless of HBsAg status. This difference can be attributed to the varying prevalence of hepatitis B and rates of drug-related hepatitis B reactivation in different regions. Combined with the above-mentioned data and the risk stratification of APASL, the reactivation rate of HBsAg (+) patients using TKI was 26%-44.1%, while that of HBsAg (-) patients was 0% (22–25). This implies that HBsAg (+) should be regarded as high risk and HBsAg (-) as low risk in CML patients receiving TKI. However, the risk stratification of CML patients receiving TKI therapy in China still lacks evidence from large sample-size studies, especially previously infected patients.

#### 2.3.2 Which indicators need to be screened?

AASLD (2018), EASL (2017), AGA (2015), and APASL (2021) all recommend CML patients be screened for HBsAg, anti-HBc, and anti-HBs in order to determine the stage of hepatitis B infection before immunosuppressive therapy. APASL also advocates screening for HBV DNA and HBsAg levels in HBsAg (+) patients as well as evaluating the degree of liver fibrosis where applicable (33–36). Based on the national statistics of China, the infection rate of hepatitis B is about 6.1% (32), and the aforementioned screening biomarkers are inexpensive. Thus, patients receiving TKI should routinely be screened for viral markers (HBsAg, anti-HBc, anti-hbs, and HBV DNA), serum liver function test (ALT, AST, coagulation function, and bilirubin levels), and liver stiffness.

#### 2.3.3 Indications for antiviral therapy

TKI competes with ATP for binding sites on ABL kinase, preventing downstream signaling pathway phosphorylation and thus inhibiting proliferation of BCR::ABL1-positive cells. However, the effects of TKIs on immune function in patients can contribute to hepatitis B reactivation, including episodes of hepatitis associated with elevated alanine aminotransferase (ALT), increased mortality from liver failure, and the progression of chronic myelogenous leukemia after reduction or discontinuation of TKIs. Therefore, the choice of people for antiviral treatment is very important. At present, there has been no standard preventive strategy established for hepatitis B patients receiving TKI treatment. However, there are usually two options.

Firstly, prophylactic antiviral therapy should be given to patients with high-risk factors:1)HBsAg (+) patients.;2)HBsAg (-)/anti-HBc (+) patients with cirrhosis or advanced liver fibrosis.

Secondly, changes in hepatitis B surface antigen and DNA were monitored dynamically, and antiviral therapy was given immediately once the following situations occurred:1)HBsAg (-)/anti-HBc (+) patients with HBsAg and/or HBV DNA seroconversion;2)HBsAg (-)/anti-HBc (+) patients with a high HBV DNA level (> 2 log of baseline) (33–36).

#### 2.3.4 How is liver fibrosis evaluated?

The assessment of liver fibrosis is of great significance for determining the diagnosis, judging the prognosis, choosing the appropriate the treatment, and monitoring treatment efficacy. Liver biopsy remains the gold standard for the diagnosis of fibrosis but is an invasive examination and relatively expensive; thus, imaging techniques are preferred. Transient elastography has been widely validated by liver stiffness measurement (LSM) as an accurate and reproducible method for predicting advanced fibrosis or cirrhosis in patients with chronic hepatitis B (CHB, chronic hepatitis B). The 2019 guidelines for transient elastography recommends that if LSM > =17.0 kPa, liver cirrhosis should be considered in hepatitis B patients with normal bilirubin levels and ALT<5×ULN; if LSM>=12.4 kPa, then advanced liver fibrosis should be considered. When bilirubin levels and ALT are within the normal range,if LSM >=12.0 kPa, a diagnosis of cirrhosis should be suspected, while LSM>=9.0 kPa indicates advanced liver fibrosis. However, the accuracy of liver biopsy is higher when the criterion of ALT>= 5×ULN is used (37, 38). The assessment of liver fibrosis can be used for more detailed stratification to guide treatment and can also serve as a reference for treatment discontinuation.

#### 2.3.5 When should the antiviral drugs be discontinued?

Patients with chronic HBV-related hepatitis are typically treated long-term with nucleoside/nucleotide analog antiviral therapy, and treatment may be discontinued based on HBV DNA and ALT levels as well as liver fibrosis assessment. However, the duration of treatment has not been established for patients initiated on prophylactic antiviral therapy. Both APASL and AASLD recommend antiviral therapy for at least 6 months after the withdrawal of immunosuppressants, whereas EASL recommends HBsAg (+) patients to receive antiviral therapy for at least 12 months after the withdrawal of immunosuppressants (33–36). Although TKI treatment is likely to be lifelong for the majority of CML patients, antiviral therapy is recommended to be continued for at least 6 months after discontinuation of the TKI, regardless of HBV DNA levels, in patients for whom a treatment-free response is expected.

#### 2.3.6 Content and frequency of monitoring after discontinuation of antiviral drugs.

HBsAg and HBV DNA levels, as well as liver function (ALT, AST, bilirubin, and coagulation profile), should be evaluated every 3 months for at least 1 year after stopping antiviral drugs. In the meantime, abdominal ultrasound or liver stiffness testing should be performed every 6 months in patients without cirrhosis. For patients with cirrhosis, lifelong medication may be considered, and reexaminations are recommended every 3 months (37).

#### 2.3.7 The choice of antiviral drugs

The 2019 guidelines for the prevention and treatment of chronic hepatitis B point out that entecavir, tenofovir, and adefovir dipivoxil have a higher safety profile and lower drug resistance rate; furthermore, long-term use can effectively reduce the risk of cirrhosis and liver cancer, and the viral response rate gradually increases over time (37). Huang et al. (39) randomly compared entecavir with lamivudine in lymphoma HBsAg(+) patients receiving an R-CHOP regimen, and the former significantly reduced the reactivation rate of hepatitis B. Likewise, a meta-analysis conducted by Zhang et al. (40)determined that entecavir (OR 0.23, 95% CI 0.10-0.49) and tenofovir (OR 0.04, 95% CI 0.00-0.43) were significantly superior to lamivudine in the absence of prophylactic antiviral therapy. Thus, prophylactic treatment with tenofovir and entecavir may be the most effective approach to preventing HBV reactivation and HBV-related morbidity and mortality in patients with chronic hepatitis B infection receiving chemotherapy sessions. In addition, these three drugs do not influence the activity of the liver drug enzyme CYP3A4, and hence, these antiviral drugs can be used in combination with TKI. However, there are no clinical studies comparing the efficacy of these three drugs in inhibiting hepatitis B reactivation. Antiviral agents can be selected according to renal function and a past history of lamivudine resistance. Entecavir is typically preferred if the patient has poor renal function. In comparison, tenofovir is preferred if lamivudine has been previously used.

#### 2.3.8 Application of hepatitis B vaccine

In 20 studies involving 1672 patients who did not receive antiviral prophylaxis, the reactivation risk was 14% in 388 patients with hepatitis B core antigen (anti-HBc) antibodies and 5.0% in 1284 patients who also had anti-HBs (41). In an experiment by Takahata et al., 46 people prepared for hematopoietic stem cell transplantation, 21 received three doses of hepatitis B vaccine prior to transplantation, and 25 did not receive any treatment, and the hepatitis B reactivation ratio was 0(0/21) vs 48%(12/25)(P=0.0003). Moreover, 11 of the 12 patients with reactivation lost anti-HBs. (42). The absence of anti-HBs may be a predictor of HBV reactivation. In addition, Chinese guidelines recommend that patients who have received hepatitis B vaccine in the past, if anti-HBs is less than 10 miU/ml, should immediately inject hepatitis B immunoglobulin, and complete vaccination according to the course of 0, 1, 6 months. Therefore, for patients taking TKI, if all markers are negative or anti-HBs <10 miU/ml, the hepatitis B vaccine can be injected (32).

## 3 Summary and prospect

The use of TKI is historic progress in the treatment of CML and has significantly enhanced survival outcomes and the quality of life of CML patients. Although the immune effects of TKI has been reported, its underlying mechanism remains elusive. In the future, larger clinical trials are warranted to investigate the potential risk of infectious diseases in CML patients receiving TKI treatment by exploring the effects of TKI on immune cells and immune function so as to develop a universal management strategy.

## Author contributions

TiY , WL and TaY contributed to the conception and design of the study. TiY wrote the original draft of the manuscript. WL and TaY supplemented the related literature and reviewed this manuscript. All authors contributed to the article and approved the submitted version.
